# An *In Vitro* Verification of the Effects of Paeoniflorin on Lipopolysaccharide-Exposed Microglia

**DOI:** 10.1155/2020/5801453

**Published:** 2020-10-12

**Authors:** Qiliang Chen, Yaojun Liu, Yuanyuan Zhang, Xinyu Jiang, Yuqin Zhang, Tetsuya Asakawa

**Affiliations:** ^1^School of Basic Medicine, Guangzhou University of Chinese Medicine, Guangzhou, Guangdong 510006, China; ^2^Research Base of Traditional Chinese Medicine Syndrome, Fujian University of Traditional Chinese Medicine, Fuzhou 350122, China; ^3^College of Pharmacy, Fujian University of Traditional Chinese Medicine, Fuzhou, Fujian 350122, China; ^4^Clinical Medical College of Acupuncture Moxibustion and Rehabilitation, Guangzhou University of Chinese Medicine, Guangzhou 510006, China; ^5^College of Acupuncture and Moxibustion, Fujian University of Traditional Chinese Medicine, Fuzhou 350122, China; ^6^Department of Neurosurgery, Hamamatsu University School of Medicine, Handayama, Hamamatsu City, Shizuoka, Japan

## Abstract

**Background:**

The neuroprotective effects of Paeoniflorin (PF) are well known. Most of the evidence was verified *in vivo*. We attempted to perform an *in vitro* verification of the effects of PF in microglia.

**Methods:**

A lipopolysaccharide- (LPS-) exposed microglia model was employed. An enzyme-linked immunosorbent assay was used to measure the levels of cytokines in the culture supernatants. A real-time polymerase chain reaction was performed to measure the mRNA expression of cytokines and M1- and M2-like genes. A western blot analysis was used to examine the expression of proteins associated with the nuclear factor-kappa B (NF-*κ*B) signaling pathway.

**Results:**

We found that the administration of PF reversed the inflammatory response induced by LPS. It downregulated proinflammatory cytokines and upregulated anti-inflammatory cytokines. This, in turn, alleviated the oxidative injuries, downregulated the expression of M1-like genes, and upregulated the expression of M2-like genes. PF can also reverse the changes in proteins associated with the NF-*κ*B signaling pathway induced by LPS.

**Conclusions:**

We provided evidence obtained *in vitro* concerning the neuroprotective effects of PF via suppressing activation of microglia, which might be associated with the NF-*κ*B signaling pathway. These findings contribute to obtaining a deeper understanding of PF, a potential new treatment for brain injuries.

## 1. Introduction

Paeoniflorin (PF), an amorphous glucoside (Supplementary Figure 1), is the major active ingredient of the *Paeoniae radix*. Its neuroprotective effects have been well documented [1–4]. However, the mechanisms underlying these effects are quite complicated and are not fully understood. It is believed that these neuroprotective effects may be associated with alleviating oxidative injury [5], ameliorating neuroinflammation via modulation of the cytokines [4], suppressing activation of the inflammation pathways [6–8], and regulating neurotrophic factors [9]. A recent study demonstrated that PF has effects on the downregulation of microRNA-135a [5]. It is, therefore, also believed that the neuroprotective effects of PF are comprehensive and involve antiapoptotic [10], anti-inflammatory, and antioxidative effects.

Although no clinical study has directly used PF in human patients, many animal studies have indicated that it may be effective in treating neuropsychological symptoms like depression [4, 9]. Nevertheless, the effects of PF on cerebral ischemia (CI) are being increasingly observed. In 2005, Liu et al. proved that the neuroprotective effects of PF are associated with activation of the adenosine A1 receptor [11]. Xiao et al. found that PF improves neurological deficits and cognitive impairments in a middle cerebral artery occlusion (MCAO) rat model [12]. Liu et al. reported that suppression of the nuclear factor-kappa B (NF-*κ*B) signaling pathway may play a role in the efficacy of PF with regard to learning dysfunction and brain damage in MCAO rats [13]. Chen et al. reported that PF may mediate CI preconditioning [14]. Later, Guo et al. found that the inhibition of MAPKs/NF-*κ*B-mediated inflammatory responses may contribute to the neuroprotective effects of PF [8]. Zhang et al. reported that PF administration inhibits neuroapoptosis in MCAO rats [10]. Tang et al. reported that PF treatment in the CI animal model may reduce CI lesions along with their associated inflammatory cells [15], as well as ameliorating neurologic deficits, suppressing neuronal apoptosis, and promoting neurogenesis [1]. Our previous study verified that regulation of the Ca^2+^/CaMKII/CREB signaling pathway plays a role in the neuroprotective effects of PF on CI reperfusion injuries [2]. All the abovementioned studies investigated the neuroprotective effects of PF from different angles but focused on verification in a CI animal model *in vivo*. *In vitro* verification at the cellular level is rare.

Microglia are an important part of a glial cell (10%–15%) in the central nervous system (CNS) and act as the primary immune cells of the brain. During the CI state, microglia are activated and induce the so-called microglia-mediated inflammatory response [16]. It has been well documented that suppression of microglia activation, as well as subsequent inhibition of the microglia-mediated inflammatory response, helps to alleviate neuroinflammatory responses and ameliorate ischemic injury. A previous study proved that the neuroprotective effects of PF were conducted by suppressing the production of the proinflammatory cytokines from activated microglial cells [17]. Based on the above evidence, we hypothesized that PF also plays a role in the suppression of microglial activation and subsequent microglia-mediated inflammatory response, which can be also observed *in vitro*. In this study, we employed a lipopolysaccharide- (LPS-) exposed microglia as a model of neuroinflammation associated with ischemic injury and investigated the neuroprotective effects of PF from the angles of inflammatory cytokines, oxidative stress, activation of microglia, and one inflammatory signaling pathway. We attempted to obtain preliminary evidence regarding the effects of PF on microglia at the cellular level, which may contribute to further understanding of the mechanisms of the neuroprotective effects of PF regarding inflammatory responses.

## 2. Methods

### 2.1. Cells and Reagents

BV-2 microglial cells (mouse, C57BL/6, brain, microglial cells, CHI Scientific Co., Ltd, Jiangyin, China) were employed in the present study. Cells were cultured in an RPMI 1640 medium with 10% FBS and a 1% penicillin-streptomycin mixed solution (Gibco Invitrogen Corporation, Carlsbad, CA, USA) at 37°C in a 5% CO_2_ incubator.

PF (purity > 98%, National Institute for the Control of Pharmaceutical and Biological Products, Beijing, China) was dissolved in dimethyl sulfoxide (DMSO) at concentrations 48 *μ*g/mL (100 *μ*m) and 96 *μ*g/mL (200 *μ*m) [18].

Cells were seeded on either 96- or 6-well plates and were randomly divided into four groups: (1) intact cells that were set as the control group; (2) an LPS-treated group with cells that were incubated with LPS (100 ng/mL, St. Louis, MO, USA) for 1 h; (3) a group with cells that were coincubated with LPS (100 ng/mL) and PF (100 *μ*m) for 1 h; and (4) a group comprised of cells that were coincubated with LPS (100 ng/mL) and PF (200 *μ*m) for 1 h. The doses of LPS and PF and the treatment time were determined comprehensively considering the doses used in the previous studies [2, 17, 19]. In our previous *in vitro* study [2], we verified that the administration of PF in 100 *μ*m and 200 *μ*m exhibited physiological activities in primary hippocampal neurons. We found that PF treatments in both 100 *μ*m and 200 *μ*m significantly increased the cellular viability, attenuated the neuron apoptosis, and reduced the intracellular Ca^2+^ concentration in N-methyl-d-aspartic acid-induced neurons [2]. We, therefore, used the 100 *μ*m as a low dose, whereas 200 *μ*m as a high dose of PF in the present study. According to the previous studies, LPS in 100 ng/mL is safe for BV-2 microglial cells and does not cause cell death [17, 19]. To eliminate the potential cytotoxicity of PF, we observed cell viability with a 3-(4,5-dimethylthiazol-2-yl)-2,5-diphenyltetrazolium bromide (MTT) reduction assay. The data from the preliminary experiments showed that under the conditions of intact control, PF (100 *μ*m), PF (200 *μ*m), LPS (100 ng/mL), PF (100 *μ*m) + LPS (100 ng/mL), and PF (200 *μ*m) + LPS (100 ng/mL), no difference of the MTT reduction was found among these groups. These results indicated that all involved compounds did not affect cell survival; thus, the cytotoxicity of PF and 100 ng/mL LPS can be ignored in this study (Supplementary Figure 2).

### 2.2. ELISAs

A standard enzyme-linked immunosorbent assay (ELISA) approach was employed to measure both the levels of cytokines and the oxidative stress parameters. After their treatment, culture supernatants were collected and centrifuged before being subject to ELISA. Interleukin (IL)-1*β*, IL-4, IL-6, and IL-10, tumor necrosis factor (TNF)-*α*, and interferon-gamma (IFN*γ*) were measured by using a mouse cytokine ELISA kit (ABclonal Biotechnology Co., Ltd, Wuhan, China). Superoxide dismutase (SOD), reactive oxygen species (ROS), malondialdehyde (MDA), and glutathione (GSH) were measured with a special ELISA kit (Nanjing Jiancheng Bioengineering Institute, Nanjing, China). All measurements were strictly performed following the manuals by a technician who was unaware of the treatments used.

### 2.3. Quantitative Reverse Transcription PCR (RT-qPCR)

A standard two-step *RT-qPCR* was employed to examine the mRNA expression of the cytokines and M1-like and M2-like genes. After treatments, the cells from the four groups were harvested and the total RNA was extracted using a non-phenol-based NeasyR Mini Kit (QIAGEN China, Shanghai, China). mRNA was reverse-transcribed into cDNA by the TransScript First-Strand cDNA Synthesis SuperMix (AT301, TransGen Biotech, Beijing, China) following the manufacturer's instructions. Briefly, total 2 *μ*g of total RNA was used as a template. Anchored Oligo (dT) 1 *μ*L was used as the primer with 2 × TS Reaction Mix 10 *μ*L and TransScript RT/RI Enzyme Mix 1 *μ*L. RNase-free water was finally added to form a 20 *μ*L reaction system for reverse transcription to obtain cDNA. The enzyme was inactivated at 85°C for 5 min. The obtained cDNA was directly used for RT-qPCR. *RT-qPCR* experiments were performed with an ABI 7900HT real-time PCR system (Applied Biosystems, Inc., Foster City, CA, USA). Subsequently, 5 *µ*L cDNA template was added to the reaction system including 1 *μ*L forward primer (10 *μ*m), 1 *μ*L reverse primer (10 *μ*m), and 2 × TransTaq HiFi PCR SuperMix II. Nuclease-free water was finally added to form a 50 *μ*L reaction system with denaturing at 94°C for 30 s, annealing at 60°C for 30 s, and primer extension at 72° for 2 min. Amplification was performed for 30 cycles. The final extension was performed at 72° for 10 min. The sequences of the PCR primers used in this study are listed as follows: CD32, forward 5′-AAT CCT GCC GTT CCT ACT GAT C-3′ and reverse 5′-GTG TCA CCG TGT CTT CCT TGA G-3′; iNOS, forward 5′-CAA GCA CCT TGG AAG AGG AG-3′ and reverse 5′-AAG GCC AAA CAC AGC ATA CC-3′; Arg1, forward 5′-TCA CCT GAG CTT TGA TGT CG-3′ and reverse 5′-CTG AAA GGA GCC CTG TCT TG-3′; Ym1/2, forward 5′-CAG GGT AAT GAG TGG GTT GG-3′ and reverse 5′-CAC GGC ACC TCC TAA ATT GT-3′; IL-1*β*, forward 5′-ATG ACC TGT TCT TTG AGG CTG AC-3′ and reverse 5′-CGA GAT GCT GCT GTG AGA TTT G-3′; IL-6, forward 5′-GAC CAA GAC CAT CCA ACT CAT C-3′ and reverse 5′-ACA TTC ATA TTG CCA GTT CTT CGT A-3′; IL-10, forward 5′-CCAAGCCTTATCGGAAATGA-3′; and reverse 5′- TTTTCACAGGGGAGAAATCG-3′; TNF-*α*, forward 5′-ATG AGC ACG GAA AGC ATG-3′ and reverse 5′-TAC GGG CTT GTC ACT CGA GTT-3′; and glyceraldehyde-3-phosphate dehydrogenase (GAPDH), forward 5′- AGC CCA GAA CAT CAT CCC TG-3′ and reverse 5′- AGC CCA GAA CAT CAT CCC TG-3′. The relative transcriptional level of the target genes was calculated with the formula 2^−ΔΔCt^ relative to their expression in the control group, and glyceraldehyde-3-phosphate dehydrogenase (GAPDH) was used as the endogenous control.

### 2.4. Western Blot Assay

A standard western blot assay was employed to measure the expression of proteins associated with the NF-*κ*B signaling pathway. Cells from the four groups were collected and lysed using the NP-40 Lysis Buffer (Beyotime, USA) containing a 1% (*v*/*v*) protease inhibitor phenylmethylsulfonyl fluoride (PMSF, Beyotime, USA) on ice and then centrifuged at 12,000 g for 15 min at 4°C. The supernatant was collected, and the protein concentration was determined via a Bicinchoninic Acid (BCA) Protein Assay Kit (Beyotime, USA). Then, proteins mixed with a loading buffer were incubated at 100°C for 6 min.

Subsequently, equal amounts of proteins were electrophoresed on sodium salt (SDS) acrylamide gels and transferred onto polyvinylidene fluoride (PVDF) membranes in the transfer buffer (glycine 2.9 g, Tris 5.8 g, SDS 0.37 g, methanol 200 ml, and adding deionized distilled water to make 1000 mL solution, PH 8.3) and tween 20 (TBST) at 400 mA and 80 V. The transfer time was IKK*β* 1.5 h, p-I*κ*B*α* 1 h, I*κ*B*α* 1 h, NF-*κ*Bp65 1 h, *β*-actin 1 h, and Histone 0.5 h, respectively. They were then blocked with 5% skim milk (Cell Signaling Technology, USA) followed by incubation with primary antibodies from the I*κ*B kinase (IKK) *β* (Rabbit, Ab124957, Abcam, USA), p-I*κ*B*α* (Mouse, #9246, CST, USA), I*κ*B*α* (Rabbit, #4812, CST, USA), NF-*κ*Bp65 (Rabbit, #8242, CST, USA), *β*-actin (Rabbit, #4967, CST, USA), and Histone (Rabbit, #4499, CST, USA) overnight at 4°C. Then, membranes were incubated with a horseradish peroxidase-conjugated secondary antibody, IgG (HRP Goat Anti-Rabbit IgG secondary antibody, #2884131; #EI15917, LuLong Biotech Co., Xiamen, China), at room temperature for 2 h. Finally, they were evaluated with ECL western detection reagents. The relative protein expression levels normalized to *β*-actin were then calculated.

### 2.5. Statistical Analysis

The statistical analysis was performed with the SPSS software (v20.0; SPSS Inc., IL, USA). One-way analysis of variance, followed by post hoc Dunnett's correction, was used for multiple comparisons. Data from at least three independent experiments are presented as the mean ± standard deviation (SD), and *p* < 0.05 was considered to indicate statistical significance.

## 3. Results

### 3.1. PF-Suppressed LPS-Induced Inflammatory Response

**Figure 1:**
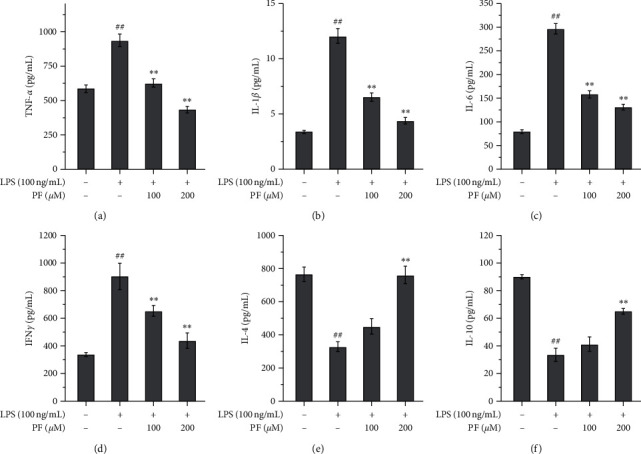
Changes in cytokines induced by the administration of PF to LPS-exposed microglia. Exposure to LPS significantly enhanced proinflammatory TNF-*α* (a), IL-1*β* (b), IL-6 (c), and IFN*γ* (d) levels, whereas the administration of PF significantly reversed these enhancements. Exposure to LPS significantly decreased anti-inflammatory IL-4 (e) and IL-10 (f) levels, whereas the administration of PF reversed these reductions. One dose of 200 *μ*m caused significant changes (*p* < 0.01). Data are shown as mean values ± SD, ## means *p* < 0.01 LPS vs. control; ^∗∗^ means *p* < 0.01, PF vs. LPS.

Figure 1 shows the changes in cytokine levels in the culture supernatants. Exposure to LPS significantly enhanced levels of TNF-*α*, IL-1*β*, IL-6, and IFN*γ* (proinflammatory cytokines), whereas it significantly decreased levels of IL-4 and IL-10 (anti-inflammatory cytokines). These LPS-induced changes in cytokine levels could be reversed with the administration of PF. For TNF-*α*, IL-1*β*, IL-6, and IFN*γ*, the levels were significantly reduced by both doses (Figures 1(a)–1(d)). For IL-4 and IL-10, only a high dose (200 *μ*m) exhibited a significant enhancement (Figures 1(e) and 1(f)). We found that a high dose of PF exhibited a stronger effect.

**Figure 2:**
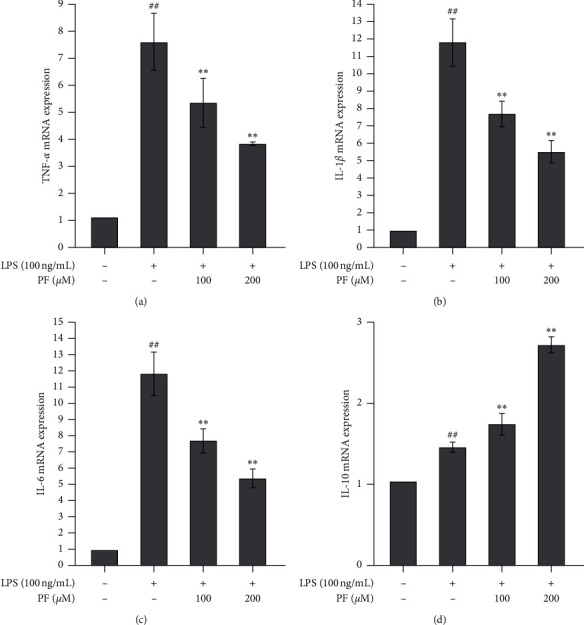
Changes in the mRNA expression of cytokines induced by the administration of PF to LPS-exposed microglia. Exposure to LPS significantly upregulated the mRNA expression of all cytokines. Administration of PF significantly downregulated the expression of TNF-*α* (a), IL-1*β* (b), and IL-6 (c) but upregulated the expression of IL-10 (d). Data are shown as mean values ± SD, ## means *p* < 0.01, LPS vs. control; ^∗∗^ means *p* < 0.01, PF vs. LPS.

Figure 2 shows the changes in the cytokines' mRNA expression within the cell extracts. Proinflammatory cytokines TNF-*α*, IL-1*β*, and IL-6 exhibited similar tendencies to those observed in the culture supernatants; namely, LPS exposure significantly upregulated their mRNA expression. Administration of PF downregulated the expression in both doses (Figures 2(a)–2(c)). The anti-inflammatory cytokine IL-10 exhibited an adverse behavior to that which we found in the culture supernatants. LPS and PF significantly upregulated its expression (Figure 2(d)). A higher dose of PF (200 *μ*m) exhibited a stronger effect.

From these data, we confirmed that PF can suppress LPS-induced inflammatory responses via the modulation of cytokines.

### 3.2. PF-Protected Microglia against LPS-Induced Oxidative Injury

**Figure 3:**
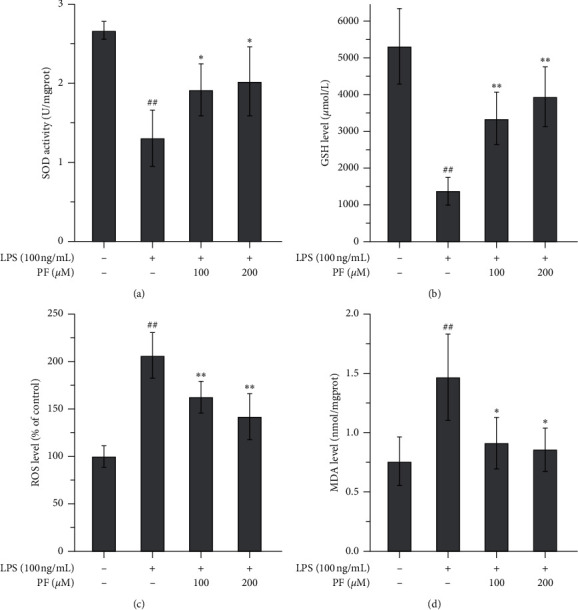
Changes of oxidative stress parameters induced by the administration of PF to LPS-exposed microglia. Exposure to LPS significantly decreased SOD (a) and GSH (b) levels and the administration of PF significantly enhanced levels of SOD and GSH. While exposure to LPS significantly enhanced ROS (c) and MDA (d) levels, the administration of PF significantly reversed these increases. Data are shown as mean values ± SD, ## means *p* < 0.01, LPS vs. control; ^∗^ means *p* < 0.05, ^∗∗^ means *p* < 0.01, PF vs. LPS.

Figure 3 shows the changes in indices concerning oxidative injury. We found that exposure to LPS decreased the levels of SOD and GSH (Figures 3(a) and 3(b)) but increased the levels of ROS and MDA (Figures 3(c) and 3(d)). These changes could be reversed by administering either a low or a high dose of PF. The high dose of PF (200 *μ*m) exhibited a stronger effect.

### 3.3. PF-Modulated LPS-Mediated Microglia Activation

**Figure 4:**
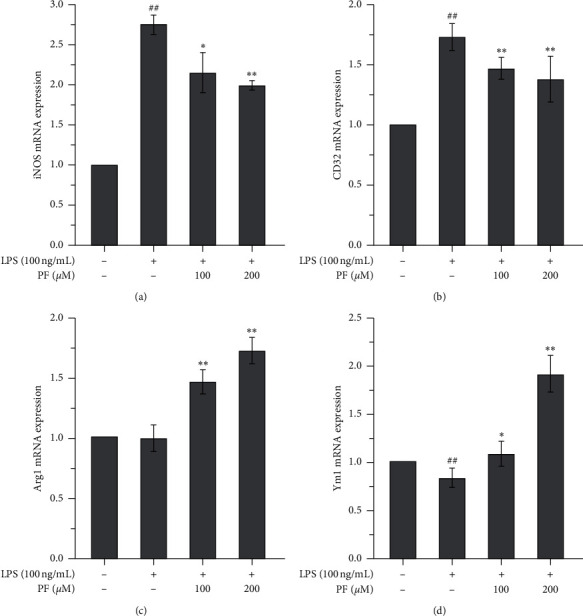
The mRNA expression of M1-like and M2-like genes affected by the administration of PF to LPS-exposed microglia. Exposure of LPS significantly upregulated the mRNA expression of M1-like genes, namely, iNOS (a) and CD32 (b), and the administration of PF significantly reversed these changes. Exposure to LPS exhibited the downregulating tendency of the mRNA's expression of M2-like genes. While Arg1 (c) did not reach a significant difference and Ym1 (d) was significantly downregulated, the administration of PF significantly reversed these decreases. Data are shown as mean values ± SD, ## means *p* < 0.01, LPS vs. control; ^∗^ means *p* < 0.05, ^∗∗^ means *p* < 0.01, PF vs. LPS.

Figure 4 shows the changes in the mRNA expression of M1-like and M2-like genes in microglia. M1-like genes exhibited an adverse behavior compared to M2-like genes. M1-like genes, including iNOS and CD32 [20], were significantly upregulated when exposed to LPS and then downregulated by either dose of PF (Figures 4(a) and 4(b)). Conversely, M2-like genes, such as Arg1 and Ym1, were downregulated when exposed to LPS (only Ym1 reached a significant difference) and were significantly upregulated by either dose of PF (Figures 4(c) and 4(d)). The changes in these genes indicated that PF may contribute to modulating the activation of microglia triggered by LPS exposure. The upregulation of M2-like genes and the downregulation of M1-like genes indicated that PF contributes to mediating the microglia expression of a protective M2 phenotype in place of a proinflammatory M1 phenotype. The high dose (200 *μ*m) of PF exhibited a stronger effect.

### 3.4. PF-Inhibited NF-*κ*B Signaling Pathways in LPS-Exposed Microglia

**Figure 5:**
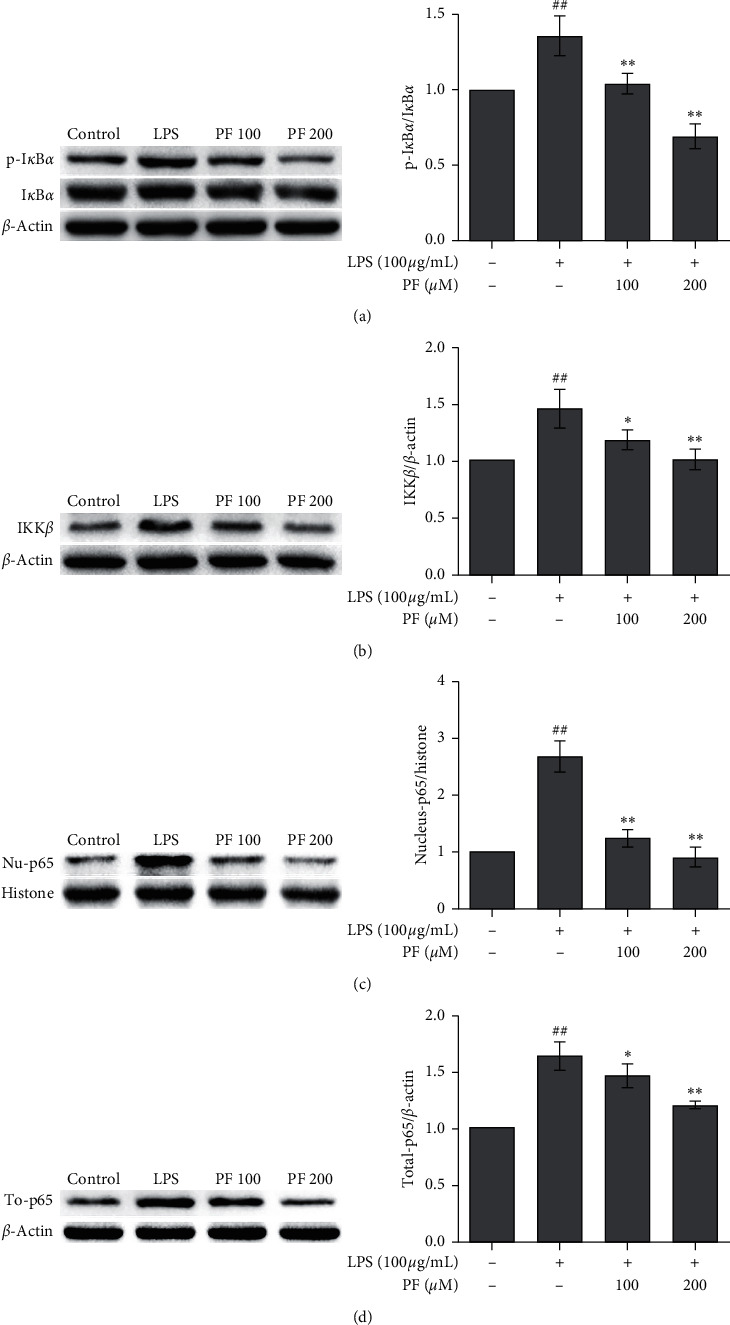
Changes in protein expression associated with the NF-*κ*B signaling pathway. Exposure to LPS may have significantly upregulated the protein expression of p-I*κ*B*α*/I*κ*B*α* (a), IKK*β* (b), nucleus NF-*κ*B (p65) (c), and total p65 (d). The administration of PF may have reversed these changes. The left column is the representative photos of western blotting analysis. Data are shown as mean values ± SD, ## means *p* < 0.01, LPS vs. control; ^∗∗^ means *p* < 0.01, PF vs. LPS.

We found that proteins associated with the NF-*κ*B signaling pathway were affected by LPS exposure and PF administration. The protein expression of p-I*κ*B*α*, IKK*β*, and NF-*κ*B (p65) was significantly upregulated by LPS exposure and then downregulated by the administration of either dose of PF. I*κ*B*α*'s protein expression exhibited the opposite behavior. The high dose (200 *μ*m) of PF has a stronger effect (Figure 5).

These data give us a hint that the suppression of the NF-*κ*B signaling pathway might play a role in the mechanisms of the effects of PF.

## 4. Discussion

In this study, we employed a BV-2 microglia cell line, established an LPS-exposed microglia model as a model of neuroinflammation in ischemic injury, and verified the neuroprotective effects of PF. We found that PF is effective in suppressing an LPS-induced inflammatory response and an oxidative injury and in modulating the activation of microglia, in which the NF-*κ*B signaling pathway might also play a role. To the best of our knowledge, this is the first study involved in verifying the neuroprotective effects of PF by suppressing the activation of microglia from different angles. We believe that these findings will be beneficial for gaining a deeper understanding of the mechanisms of PF.

Microglia are critical immune cells in the CNS. Commonly, microglia are in a resting state; however, they can be stimulated and activated by many factors. Different stimuli might trigger microglial expression of two phenotypes, namely, M1 and M2 [20]. Once microglia are activated by ischemic injury, inflammatory cytokines like IFN*γ*, or toxicants like LPS, microglia will express an M1-like gene. M1 microglia are regarded as proinflammatory and associated with proinflammatory mediators, inflammatory reactions, and brain injuries. Conversely, once microglia are stimulated by some cytokines like IL-4 or IL-13, microglia will express an M2-like gene. M2 microglia are regarded as *healing cells* and are associated with anti-inflammatory properties, tissue repair, etc. [21]. In this study, we employed a widely used LPS exposure method to mimic a harmful ischemic stimulation in the microglia. We found that classical proinflammatory cytokines, such as TNF-*α*, IL-1*β*, and IL-6, were upregulated not only in the cell matrix (Figures 1(a)–1(c)) but also in the cell (Figures 2(a)–2(c)). Importantly, the IFN*γ* level in the supernatants was also upregulated (Figure 1(d)), which shows that it is a stimulant for developing M1 microglia. However, adverse changes in anti-inflammatory cytokines were observed. Levels of IL-4 and IL-10 in the supernatants were significantly decreased by exposure to LPS (Figures 1(e) and 1(f)), but we cannot understand the changes to IL-10 in the cells themselves as it was also upregulated by LPS exposure (Figure 2(d)). Further investigation into whether this is a compensative response or not is needed. LPS also induced oxidative injuries, with decreasing levels of SOD and GSH (Figures 3(a) and 3(b)) and increasing levels of ROS and MDA (Figures 3(c) and 3(d)). Interestingly, LPS exposure upregulated the M1-like proinflammatory genes iNOS and CD32 (Figures 4(a) and 4(b)) and downregulated the M2-like healing genes Arg1 and Ym1 (Figures 4(c) and 4(d)). We believe that the upregulation of M1-like genes along with the proinflammatory cytokines must promote more microglia to be an M1 phenotype and trigger an inflammatory reaction. All these data suggest that LPS is a good stimulant for mimicking a harmful ischemic stimulus *in vitro*, in order to activate the microglia.

We found that administering PF can reverse the inflammatory response induced by LPS and that a higher dose (200 *μ*m) exhibits better efficacy than a low dose (100 *μ*m). It downregulated the proinflammatory cytokines in the cell matrix (Figures 1(a)–1(d)) and the cell (Figures 2(a)–2(c)). Interestingly, for anti-inflammatory cytokines, changes induced by PF are similar in both the cell matrix and the cell. PF may upregulate such anti-inflammatory cytokines (Figures 1(e) and 1(f) and Figure 2(d)) and also contributes to reversing oxidative injury caused by LPS. The levels of SOD and GSH increased, while the levels of ROS and MDA decreased (Figure 3). Meanwhile, PF downregulated the proinflammatory M1-like genes and upregulated the healing M2-like genes, which prompted the microglia to express a protective M2 phenotype rather than a proinflammatory M1 phenotype. These results are in agreement with the previous analogous studies using *in vivo* and *in vitro* models of other diseases. In terms of *in vivo* studies, Luo et al. found that the expression of proinflammatory M1 cytokines, such as IL-1*β*, IL-6, and TNF-*α*, was significantly downregulated, whereas the expression of anti-inflammatory cytokines, such as IL-10, was upregulated by PF treatment in hippocampal tissues of vascular dementia rat models [22]. Zhou et al. demonstrated that PF remarkably decreased the expression of IL-1*β* and TNF-*α* in the spinal cord segments of chronic constrictive injury rat models [23]. Fan et al. reported that the expression of IL-1*β* in the spinal cords of postoperative pain mouse models was suppressed by PF treatment [24]. In an *in vitro* study using beta-amyloid exposed BV-2 microglial cells, Liu et al. found that PF suppressed the upregulation of IL-1*β*, IL-6, and TNF-*α* induced by the exposure of beta-amyloid 1–42 in microglia; thereby, the activation of microglia was also inhibited [25]. With the evidence above, we verified the neuroprotective effects of PF, which may be associated with the regulation of cytokines and the reduction of oxidative injury modulated by microglia activation.

As for the inflammatory signaling pathway, we found that LPS induced the upregulation of p-I*κ*B*α* (Figure 5(a)), IKK*β* (Figure 5(b)), nucleus NF-*κ*B (p65) (Figure 5(c)), and total (p65) (Figure 5(d)). These results agree with the changes in proteins associated with the NF-*κ*B signaling pathway induced by LPS on other cell lines as described in earlier studies [26–28]. The administration of PF can reverse these changes, indicating that the effects of PF on microglia may be associated with the NF-*κ*B signaling pathway. Early in 2012, Guo et al. reported that PF exhibits neuroprotective effects with regard to ischemic damage *in vivo* [8]. Luo et al. also found that PF treatment significantly suppressed the mTOR/NF-*κ*B proinflammatory pathway and activated the PI3K/Akt anti-inflammatory pathway in hippocampal tissues of hypoperfusion-induced vascular dementia rat models [22]. An *in vitro* study also verified that the NF-*κ*B signaling pathway plays a role in the PF effects. They found that the PF administration significantly suppressed the nuclear translocation of p65 and phosphorylation of I*κ*B*α* in beta-amyloid 1–42 exposed microglia [25]. Our data are analogous to their results. The results of this study strengthen the evidence that the NF-*κ*B signaling pathway may play a role in the effects of PF. Further verification using agonists/antagonists in the NF-*κ*B signaling pathway will be included in our future study.

Taken together, in this study, we used an LPS exposure microglia model to verify the neuroprotective effects of PF. We found that the administration of PF can reverse the inflammatory response induced by LPS. It helps to downregulate proinflammatory cytokines and upregulate anti-inflammatory cytokines, alleviate oxidative injury, downregulate the expression of M1-like genes, and upregulate the expression of M2-like genes. High doses of PF exhibited better efficacy than low doses. Thus, the neuroprotective effects of PF were confirmed; furthermore, we found that PF can also reverse the changes in proteins associated with the NF-*κ*B signaling pathway induced by LPS, which provides a hint concerning the role of the NF-*κ*B signaling pathway.

## 5. Conclusion

The present study provided evidence gained *in vitro* concerning the neuroprotective effects of PF via suppressing the activation of microglia. We performed a cellular level verification and found that PF may suppress the inflammatory response, alleviate oxidative injury, and modulate the activation of microglia. These effects are associated with the NF-*κ*B signaling pathway. The findings contribute to obtaining a deeper understanding of the underlying mechanisms of PF, a potential new treatment for brain injuries.

## Data Availability

The data used to support the findings of this study are included within the article.

## Supplementary Materials

Supplementary Materials Supplementary Figure 1: the chemical structure of PF. Supplementary Figure 2: results of the MTT assay.

## References

[1] Ko C.-H., Huang C.-P., Lin Y.-W., Hsieh C.-L. (2018). *Paeoniflorin* has anti-inflammation and neurogenesis functions through nicotinic acetylcholine receptors in cerebral ischemia-reperfusion injury rats. *Iranian Journal of Basic Medical Sciences*.

[2] Zhang Y., Qiao L., Xu W. (2017). Paeoniflorin attenuates cerebral ischemia-induced injury by regulating Ca^2+^/CaMKII/CREB signaling pathway. *Molecules*.

[3] Li Y., Lu Y., Hu J. (2016). Pharmacokinetic comparison of scutellarin and paeoniflorin in sham-operated and middle cerebral artery occlusion ischemia and reperfusion injury rats after intravenous administration of xin-shao formula. *Molecules*.

[4] Li J., Huang S., Huang W. (2017). Paeoniflorin ameliorates interferon-alpha-induced neuroinflammation and depressive-like behaviors in mice. *Oncotarget*.

[5] Zhai A., Zhang Z., Kong X. (2019). Paeoniflorin alleviates H_2_O_2_-induced oxidative injury through down-regulation of MicroRNA-135a in HT-22 cells. *Neurochem Res*.

[6] Zhang J., Dou W., Zhang E. (2014). Paeoniflorin abrogates DSS-induced colitis via a TLR4-dependent pathway. *American Journal of Physiology-Gastrointestinal and Liver Physiology*.

[7] Yu J., Xiao Z., Zhao R., Lu C., Zhang Y. (2017). Paeoniflorin suppressed IL-22 via p38 MAPK pathway and exerts anti-psoriatic effect. *Life Sciences*.

[8] Guo R. B., Wang G. F., Zhao A. P., Gu J., Sun X. L., Hu G. (2012). Paeoniflorin protects against ischemia-induced brain damages in rats via inhibiting MAPKs/NF-kappaB-mediated inflammatory responses. *PLoS One*.

[9] Hu M.-Z., Wang A.-R., Zhao Z.-Y., Chen X.-Y., Li Y.-B., Liu B. (2019). Antidepressant-like effects of paeoniflorin on post-stroke depression in a rat model. *Neurological Research*.

[10] Zhang Y., Li H., Huang M. (2015). Paeoniflorin, a monoterpene glycoside, protects the brain from cerebral ischemic injury via inhibition of apoptosis. *The American Journal of Chinese Medicine*.

[11] Liu D.-Z., Xie K.-Q., Ji X.-Q., Ye Y., Jiang C.-L., Zhu X.-Z. (2005). Neuroprotective effect of paeoniflorin on cerebral ischemic rat by activating adenosine A1 receptor in a manner different from its classical agonists. *British Journal of Pharmacology*.

[12] Xiao L., Wang Y. Z., Liu J., Luo X. T., Ye Y., Zhu X. Z. (2005). Effects of paeoniflorin on the cerebral infarction, behavioral and cognitive impairments at the chronic stage of transient middle cerebral artery occlusion in rats. *Life Sciences*.

[13] Liu J., Jin D.-Z., Xiao L., Zhu X.-Z. (2006). Paeoniflorin attenuates chronic cerebral hypoperfusion-induced learning dysfunction and brain damage in rats. *Brain Research*.

[14] Chen D.-M., Xiao L., Cai X., Zeng R., Zhu X.-Z. (2006). Involvement of multitargets in paeoniflorin-induced preconditioning. *Journal of Pharmacology and Experimental Therapeutics*.

[15] Tang N.-Y., Liu C.-H., Hsieh C.-T., Hsieh C.-L. (2010). The anti-inflammatory effect of paeoniflorin on cerebral infarction induced by ischemia-reperfusion injury in Sprague-Dawley rats. *The American Journal of Chinese Medicine*.

[16] Yang X., Asakawa T., Han S. (2016). Neuroserpin protects rat neurons and microglia-mediated inflammatory response against oxygen-glucose deprivation- and reoxygenation treatments in an *In Vitro* study. *Cellular Physiology and Biochemistry*.

[17] Nam K.-N., Yae C. G., Hong J.-W., Cho D.-H., Lee J. H., Lee E. H. (2013). Paeoniflorin, a monoterpene glycoside, attenuates lipopolysaccharide-induced neuronal injury and brain microglial inflammatory response. *Biotechnology Letters*.

[18] Qiu J., Chen M., Liu J. (2016). The skin-depigmenting potential of Paeonia lactiflora root extract and paeoniflorin: *in vitro* evaluation using reconstructed pigmented human epidermis. *International Journal of Cosmetic Science*.

[19] Wang S., Luo Q., Fan P. (2019). Cannabisin F from hemp (*cannabis sativa*) seed suppresses lipopolysaccharide-induced inflammatory responses in BV2 microglia as SIRT1 modulator. *International Journal of Molecular Sciences*.

[20] Saqib U., Sarkar S., Suk K., Mohammad O., Baig M. S., Savai R. (2018). Phytochemicals as modulators of M1-M2 macrophages in inflammation. *Oncotarget*.

[21] Orihuela R., McPherson C. A., Harry G. J. (2016). Microglial M1/M2 polarization and metabolic states. *British Journal of Pharmacology*.

[22] Luo X.-Q., Li A., Yang X. (2018). Paeoniflorin exerts neuroprotective effects by modulating the M1/M2 subset polarization of microglia/macrophages in the hippocampal CA1 region of vascular dementia rats via cannabinoid receptor 2. *Chinese Medicine*.

[23] Zhou D., Zhang S., Hu L. (2019). Inhibition of apoptosis signal-regulating kinase by paeoniflorin attenuates neuroinflammation and ameliorates neuropathic pain. *Journal of Neuroinflammation*.

[24] Fan Y.-X., Hu L., Zhu S.-H. (2018). Paeoniflorin attenuates postoperative pain by suppressing matrix Metalloproteinase-9/2 in mice. *European Journal of Pain*.

[25] Liu H., Wang J., Wang J., Wang P., Xue Y. (2015). Paeoniflorin attenuates A*β*1-42-induced inflammation and chemotaxis of microglia in vitro and inhibits NF-*κ*B- and VEGF/Flt-1 signaling pathways. *Brain Research*.

[26] Lai J.-L., Liu Y.-H., Liu C. (2017). Indirubin inhibits LPS-induced inflammation via TLR4 abrogation mediated by the NF-*k*B and MAPK signaling pathways. *Inflammation*.

[27] Zhao L., Li M., Sun K., Su S., Geng T., Sun H. (2019). Hippophae rhamnoides polysaccharides protect IPEC-J2 cells from LPS-induced inflammation, apoptosis and barrier dysfunction in vitro via inhibiting TLR4/NF-*κ*B signaling pathway. *International Journal of Biological Macromolecules*.

[28] Shi L., Fang B., Yong Y. (2019). Chitosan oligosaccharide-mediated attenuation of LPS-induced inflammation in IPEC-J2 cells is related to the TLR4/NF-*κ*B signaling pathway. *Carbohydrate Polymers*.

