# Supplementary material for: An In Vitro Verification of the Effects of Paeoniflorin on Lipopolysaccharide-Exposed Microglia
Source: Evid Based Complement Alternat Med. 2020 Oct 12;2020:5801453. doi: 10.1155/2020/5801453 (PMC7576368; doi:10.1155/2020/5801453)
Supplement: Supplementary Materials — Supplementary Figure 1: the chemical structure of PF. Supplementary Figure 2: results of the MTT assay. [file 5801453.f1.pdf]

Supplementary Materials

Supplementary Figure 1. The chemical structure of PF

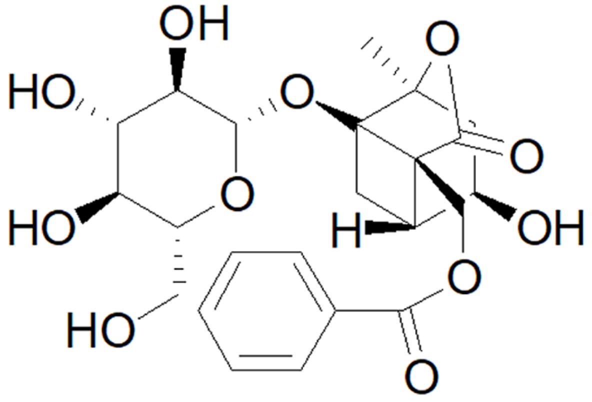

Supplementary Figure 2. Results of the MTT assay

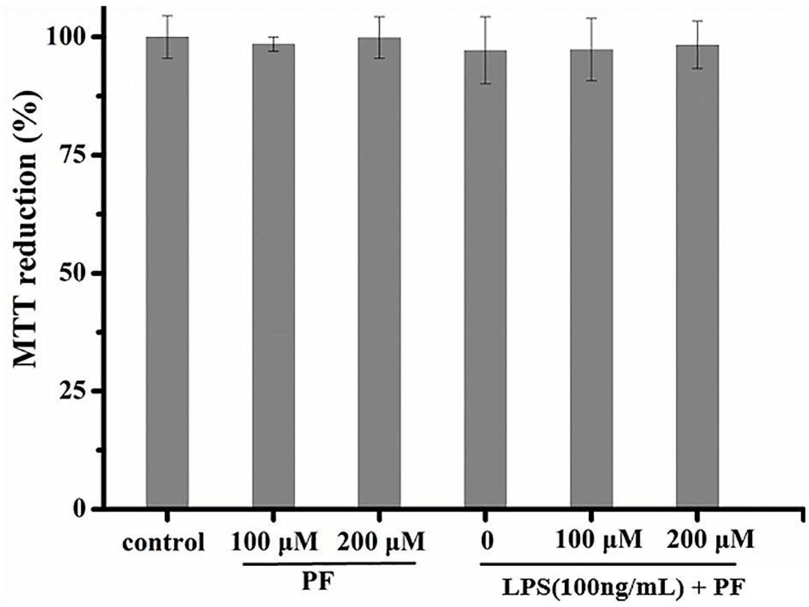

No difference in the MTT reduction was found between the control and the PF groups with or without LPS administration.

These data eliminated the potential cytotoxicity of PF treatments and LPS (100 ng/mL) in BV-2 microglia.
